# Neutrophil-to-Lymphocyte Ratio (NLR) Predicts PD-1 Inhibitor Survival in Patients with Metastatic Gastric Cancer

**DOI:** 10.1155/2021/2549295

**Published:** 2021-12-28

**Authors:** Miaomiao Gou, Tongtong Qu, Zhikuan Wang, Huan Yan, Yanhai Si, Yong Zhang, Guanghai Dai

**Affiliations:** ^1^Medical Oncology Department, The First Medical Center, Chinese PLA General Hospital, Fuxing Road 28, Haidian District, Beijing, China 100853; ^2^Medical Oncology Department, The Second Medical Center, Chinese PLA General Hospital, Fuxing Road 28, Haidian District, Beijing, China 100853

## Abstract

**Background and Aims:**

Biomarkers for systemic inflammation have been introduced into clinical practice for risk-rating in cancer patients' treatment. This study is aimed at confirming the prognostic role of the neutrophil-to-lymphocyte ratio (NLR) as an effective biomarker for patients with metastatic gastric cancer (MGC) receiving anti-PD-1 agents.

**Method:**

Patients with MGC who received anti-PD-1 treatment at the Chinese PLA General Hospital between January 2016 and November 2020 were reviewed. The study analyzed the association of NLR and overall survival (OS) or progression-free survival (PFS) and antitumor response rate with PD-1 inhibitors.

**Results:**

137 patients were included in the final analysis. The area under the curve value of NLR for 6-month OS was 0.71. The best cut-off value for NLR was 3.23. NLR < 3.23 was associated with longer OS (HR = 0.38, 95% CI, 0.26-0.57, *p* < 0.001) and PFS (HR = 0.42, 95% CI, 0.29-0.62, *p* < 0.001) in patients with MGC. No significant difference was observed in the objective response rate (ORR) (35.8% vs. 28.6%, *p* = 0.377) and disease control rate (DCR) (86.4% vs. 78.6%, *p* = 0.229) in the NLR < 3.23 group and in the NLR ≥ 3.23 group, respectively. Univariate analysis and multivariate analysis found that NLR was an independent prognosis biomarker for PFS and OS.

**Conclusions:**

Pretreatment elevated NLR was significantly associated with inferior PFS and OS in patients with MGC who received anti-PD-1 inhibitors. Clinicians need to consider patients with elevated NLR for decisions on immunotherapy strategy.

## 1. Introduction

Gastric cancer (GC) is the second most common cause of death amongst cancers, according to the statistics of 2015 in China [1]. The standard of care for patients with her-2 negative metastatic gastric cancer (MGC) is a double or triple regimen of chemotherapy [2]. The overall survival for MGC is poor, with a mean survival of 12-14 months [3]. Immune checkpoint inhibitors (ICI) have shown promising anticancer activity in a variety of cancers such as melanoma [4], non-small lung cancer [5], renal cell cancer [6], esophagus cancer [7], liver cancer [8], and gastric cancer [9]. FDA-approved Nivolumab and Pembrolizumab (two fully human IgG4 monoclonal antibodies blocking the programmed death-1 (PD-1) receptor) are considered the mainstay treatment for patients with MGC who failed to respond to chemotherapy [10, 11]. However, debates have recently arisen over the timing of anti-PD-1 antibody administration for late-stage gastric cancer. Pembrolizumab was shown to be nonsuperior to chemotherapy in the first- and second-line treatments in the clinical trials [12, 13]. In contrast, another anti-PD-1 agent called Nivolumab, when combined with chemotherapy as first line, was more effective than the standard treatment in AGC and MGC, irrespective of tumor programmed death ligand-1 (PD-L1) expression [14]. PD-L1 expression and other potential biomarkers, including tumor-infiltrating lymphocyte (TIL), microsatellite instability (MSI), tumor mutation burden (TMB), and Epstein–Barr virus (EBV), are predictive markers for ICI treatment outcomes in gastric cancer [15–17]. However, in most cases, assessing these potential biomarkers is expensive, time-consuming, or not routinely feasible during therapy. Therefore, they cannot be applied for an upfront selection of patients.

Tumor-induced system inflammatory response was confirmed as an effective prognostic biomarker in many cancers [18–23]. The response includes leukocyte, neutrophil, and monocyte counts; neutrophil-to-lymphocyte ratio (NLR); monocyte-to-lymphocyte ratio (MLR); and platelet-to-lymphocyte ratio (PLR). NLR has been recognized as a prognostic predictor of survival in patients undergoing surgery with gastric cancer at the early stage [24] or patients with late stage treated with chemotherapy or Nivolumab monotherapy [21, 25–31]. To our knowledge, however, the predictive value of NLR for MGC patients with anti-PD-1 antibodies in combination with other therapies has not been widely studied. Therefore, it is of interest to investigate whether NLR can predict survival outcomes in patients with MGC treated with anti-PD-1.

## 2. Patients and Method

### 2.1. Study Patients

Medical records from MGC patients who received PD-1 inhibitor in the Oncology Department of the Chinese PLA General Hospital between January 2016 and November 2020 were retrospectively reviewed. The study adhered to the Declaration of Helsinki and was approved by the hospital's ethics committee. Patients who received any PD-L1 inhibitor and anti-CTLA4 treatment were excluded. The inclusion criteria were as follows: (1) pathologically diagnosed MGC, (2) complete clinical characteristics available, (3) at least one measurable lesion and confirmed response outcome, (4) complete blood cell count before the initiation of anti-PD-1 inhibitor, and (5) acquired survival status. All the data was obtained from electronic clinical chart in our oncology department. The baseline NLR was defined as the neutrophil count divided by the lymphocyte count. The receiver operating characteristic analyses for predicting 6-month and 12-month progression-free survival (PFS) by the NLR was applied to identify an appropriate cut-off value for NLR according to previous studies [30]. The patients were then divided into the low NLR group (<cut-off value of NLR) and high NLR group (≥cut-off value of NLR). The studies involving human participants were reviewed and approved by the ethics committee of the Chinese People's Liberation Army General Hospital (PLAGH).

### 2.2. Treatment

PD-1–targeting antibodies included Nivolumab at a dose of 3 mg/kg, Pembrolizumab at 200 mg intravenously, Sintilimab (domestic anti-PD-1 agents) at 200 mg per cycle, or Toripalimab (another anti-PD-1 agent) as a stable 240 mg dosage, which was given alone or followed by cytotoxicity regime or other angiogenesis inhibitors. Angiogenesis inhibitors can be small-molecule tyrosine kinase inhibitors (TKI) such as Apatinib or monoclonal antibodies such as Bevacizumab. The combined regimen was based on the patient's condition and preference. All the patients signed informed consent for treatment.

### 2.3. Assessment

The study's primary end-point was to assess the prognostic significance on progression-free survival (PFS) and overall survival (OS) with different pretreatment NLR levels. PFS was defined from the date of any treatment initiation until the date of either the first progression or death. OS was calculated from the initiation of each line treatment until the date of death due to any cause. Depending on the drug regimen, treatment efficacy was assessed using the Response Evaluation Criteria in Solid Tumors (RECIST). The response was graded into four classes: complete response (CR), partial response (PR), stable disease (SD), and progressive disease (PD). Objective response was defined as CR plus PR, while disease control was defined as CR, PR, or SD.

### 2.4. Statistical Analysis

All statistical analyses were performed using SPSS for Windows version 20.0 (SPSS, Inc., Chicago, IL). The difference of category variables of characteristics among the low NLR group and high NLR was compared by *χ*^2^ tests. Survival curves of PFS and OS were estimated by the Kaplan–Meier method. The log-rank test was used to compare OS and PFS between groups when the patients were stratified into quartiles based on NLR or the cut-off values. Prognostic factors for OS and PFS were performed by univariate and multivariate analyses. R package survival ROC, Version 1.03, was used to calculate the cut-off of NLR [32]. The level of significance was set to *p* < 0.05.

## 3. Result

A total of 137 patients were enrolled in the analysis following the specified criteria. Of these, 98 were males and 39 females, with a median age of 59 years. Among them, 71 patients were administered immunotherapy as a first line while the rest received immunotherapy as the second or further line. 92 patients had received immunotherapy combined with chemotherapy, whereas 45 patients had undertaken immunotherapy alone or with angiogenesis inhibitors. The median neutrophil-to-lymphocyte ratio (NLR) in all patients was 4.65 (range, 0.72–79, 95% CI 3.34-5.96). The median PFS was 5.2 m, and OS was 10.9 m among 137 patients.

**Figure 1:**
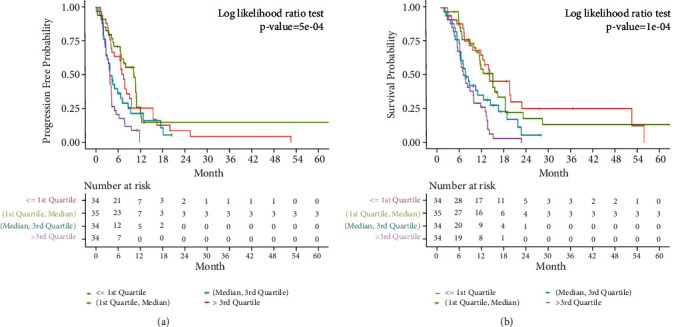
Progression-free survival (PFS) (a) and overall survival (OS) (b) in NLR quartile.

**Figure 2:**
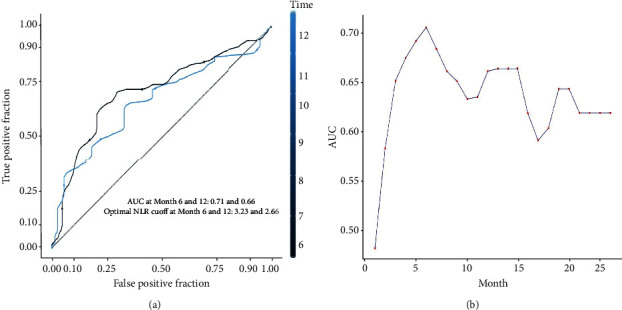
(a) Receiver operating characteristic curves for predicting 6-month and 12-month PFS by the neutrophil-to-lymphocyte ratio (AUC areas under the curve are 0.71 and 0.66). (b) AUC value by PFS time.

Patients were plotted as a cohort by quartiles based on NLR. The significant difference of PFS and OS is shown in the patients' cohort stratified into quartiles of NLR (*p* < 0.01; Figure 1). The optimal cut-off value of NLR was 3.23(AUC = 0.71, *p* < 0.001) (Figure 2). Patients were divided into two groups: the NLR < 3.23 group (low NLR) and the NLR ≥ 3.23 group (high NLR). A summary of the patients' characteristics in the NLR < 3.23 group (81 cases) and the NLR ≥ 3.23 group (56 case) is shown in Table 1. None of the clinical features in the two groups was compared with the difference in terms of gender, age, Eastern Cooperative Oncology Group Performance Status (ECOG PS), PD-L1 expression status, primary tumor site, histological differentiation, number of metastatic sites, presence of liver metastasis, smoking and drinking habit, and anti-PD-1 treatment line and type (Table 1).

We recorded an objective response in 29 of 81 patients (35.8%) with 2 complete responses (CR) in the NLR < 3.23 group and 16 out of 56 patients (28.6%) in the NLR ≥ 3.23 group. The confirmed disease control rate (DCR) was 86.4% in the low NLR group and 78.6% in the counterpart group. No statistical difference of ORR and DCR was observed in the two groups (*p* = 0.377 and *p* = 0.229, respectively) (Table 2).

**Figure 3:**
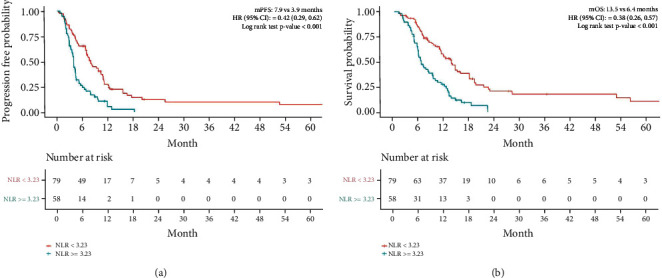
Progression-free survival (PFS) (a) and overall survival (OS) (b) in NLR based on the NLR cut-off value.

As shown in Figure 3, the PFS in the low NLR group was 7.9 m while the PFS in the high NLR group was 3.9 m (HR = 0.42, 95% CI 0.29-0.62, *p* < 0.001). Similarly, OS was 13.5 m in the low NLR group and 6.3 m in the high NLR group (HR = 0.38, 95% CI, 0.26-0.57, *p* < 0.001). The risk of death was significantly lowered by 62% in the low NLR group than in the high NLR group. The curves for both groups were well separated over time.

Clinical variables for prognosis prediction were evaluated with univariate and multivariate analyses. Univariate analysis revealed that patients receiving immunotherapy in the first line had significantly higher PFS and OS than patients with immunotherapy in the second line or further line. Anti-PD-1 plus chemotherapy led to longer PFS and OS than ICI monotherapy or with antiangiogenic drugs given to patients (*p* < 0.05). Patients with ECOG PS ≥ 2 were associated with poorer PFS (HR = 1.96, 95% CI 1.14-3.37, *p* = 0.012) but not OS (HR = 1.18, 95% CI 0.67-2.08, *p* = 0.557). High pretreatment NLR was associated with lower PFS and OS. Multivariate analysis suggested that NLR was a significant and independent prognosis biomarker for PFS and OS (Tables 3 and 4).

## 4. Discussion

Previous studies had extensively demonstrated that pretreatment NLR is a reliable prognostic marker for metastatic melanoma patients undergoing Ipilimumab or Nivolumab treatment [23, 33]. A meta-analysis including 738 ICI-treated patients with NSCLC or melanoma or advanced GU cancer showed that a high NLR was associated with poorer outcomes [34]. Currently, only two studies focused on NLR changes after Nivolumab monotherapy or baseline NLR prior to Nivolumab for gastric cancer in the third or further lines [26, 28]. Several trials had shown that Nivolumab combined with chemotherapy was superior to chemotherapy alone in the first line concerning PFS and OS [9, 35]. Anti-PD-1 monotherapy or combined therapy had been prevailingly applied in the first or second line for AGC and MGC patients. However, anti-PD-1 monotherapy is still not recommended by the National Comprehensive Cancer Network (NCCN) or domestic guidelines. We observed that not all patients were suitable for treatment with anti-PD-1 monotherapy. Due to this, it is necessary to investigate the convenient potential biomarker for the identification of cancer patients who are prescribed with anti-PD-1 in any line or any combination at high risk.

To the best of our knowledge, our study showed for the first time that NLR is an independent predictor of which MGC patients are more likely to benefit from anti-PD-1 treatment. Patients in the low NLR group had a longer survival time than those in the high NLR group, which was consistent with other studies [24, 30, 31, 36–38]. Murakami et al. had reported that the median survival times were 9.1 months in the NLR high group and 17.1 months in NLR low group (*p* < 0.0001) among patients with unresectable GC. Elevated NLR was also associated with worse DSS (HR, 1.11; 95% CI, 1.08–1.14; *p* < 0.01) in resectable GC from the study of Wang et al. [39]. A systematic review and meta-analysis also further revealed an association of high neutrophil-lymphocyte ratios with older age, male gender, and lower 5-year overall survival in gastric cancer patients submitted to curative resection [38]. Why NLR is associated immunotherapy outcome may be attributed to the following reasons: inflammatory reactions induced by tumors generate a cancer-related inflammatory microenvironment resistant to the immune monitor [40]. Neutrophils are a systemic inflammatory index because they suppress lymphocytes' immune activity by producing chemokines and cytokines [41]. This may explain the negative response to immunotherapy when there is a high level of neutrophils. On the other hand, the lymphocyte count represents a measure of lymphocyte infiltration around the tumor tissues, which has already been reported in association with prognosis in solid cancers [42, 43]. A lower NLR stands for relative lymphocyte dominance and reflects the unique properties of favorable inflammatory microenvironment for a subsequent antitumor immunologic reaction. Therefore, in view of these considerations, we suggest that patients with low NLR are prone to have better inflammatory responses and prognoses. Thus, careful attention must be paid when PD-1 inhibitors are given to patients with high NLR levels.

The cut-off value in our study was in accordance with ROC analysis by six-month survival. The optimal candidate cut-off value was 3.23 for NLR. The values of cut-off NLR are from 2.5 to 4 reported in gastric cancer among other studies [27, 36, 37, 44]. However, there was no standard for selecting the cut-off value. Some studies had applied the receiver operating characteristic (ROC) curve for estimating the cut-off value [21, 26, 28, 45–47]. The median value of NLR was also used as the cut-off of NLR in the study [24, 48, 49]. Furthermore, in our study, we found that NLR had the highest area under the curve for six-month other than one-year survival. In contrast, NLR had the highest area under the curve for one year among patients with unresectable GC in this study [30]. So it is of controversy, and we adopted the cut-off value with highest area under curve in ROC analysis in our study according to previous reports.

Interestingly, in our study, the low NLR group had a nonsuperior disease control rate (DCR) and ORR than the high NLR group (*p* > 0.05). A few clinical studies have shown the association between NLR and response to chemotherapy or immunotherapy in solid cancers [50]. In contrast with our findings, a study found that the low NLR group had a significantly higher disease control rate than the high NLR group in metastatic advanced gastric cancer response to chemotherapy [49]. However, although there was no statistical difference in ORR and DCR in our study, ORR was numerally higher in the low NLR group than the high NLR group (35.8% vs. 28.6%). The current study adds an additional component to our understanding of the predictive value of NLR in immunotherapy.

Univariate and multivariate analyses also showed that NLR is an independent prognostic biomarker for MGC patients exposed to anti-PD-1 therapy. Nowadays, PD-L1 expression, TMB or TIL, and microsatellite instability (MSI) have been identified as potential biomarkers in many solid tumors [51–59]. However, TMB and TIL evaluations are not routinely performed in clinical practice. Instead, NLR can be tested with a standard blood test and is particularly advantageous as a biomarker because of its availability and cost-effectiveness.

Limitations of this study included the small sample size with retrospective design and absence of an external validation series for the cut-off value of PLR. This observational study was based on a single institution which may cause selection bias. It further stimulates us to conduct more studies for the validation of predictive value of NLR for the response to treatment with the checkpoint inhibitors in patients with MGC.

## 5. Conclusion

In summary, we showed that pretreatment NLR is significantly and independently associated with PFS and OS in patients with MGC receiving PD-1 inhibitors. Therefore, NLR should be considered as an aid for decisions on treatment strategy and as an effective prognostic predictor.

## Figures and Tables

**Table 1:** The characteristics of the analyzed patients in different groups.

Characteristics	NLR < 3.23		NLR ≥ 3.23		*p* value
	*N*	%	*N*	%	
No. patients	81		56		
Gender *n* (%)					
Male	59	72.8%	39	69.6%	0.685
Female	22	27.2%	17	30.4%	
Age median = 59*n* (%)					
<59	35	43.2%	21	37.5%	0.505
≥59	46	56.8%	35	62.5%	
PD-L1 *n* (%)					
Positive	14	17.3%	11	19.6%	0.227
Negative	14	17.3%	15	26.8%	
Unknown	53	65.4%	30	53.6%	
ECOG PS *n* (%)					
0	26	32.1%	10	17.9%	0.508
1	43	53.1%	43	76.8%	
≥2	12	14.8%	3	5.4%	
Tumor_location *n* (%)					
Cardia	25	30.9%	14	25.0%	0.448
Body/fundus	50	61.7%	37	66.1%	
Pylorus	6	7.4%	5	8.9%	
Histological_differentiation *n* (%)					
Poorly	40	49.4%	31	55.4%	0.473
Moderately	37	45.7%	23	41.1%	
Well	4	4.9%	2	3.6%	
No. of metastasis organs *n* (%)					
<2	24	29.6%	11	19.6%	0.189
≥2	57	70.4%	45	80.4%	
Liver metastasis *n* (%)					
Yes	49	60.5%	31	55.4%	0.55
No	32	39.5%	25	44.6%	
Smoking_history *n* (%)					
Smoke	30	37.0%	29	51.8%	0.088
Never smoked	51	63.0%	27	48.2%	
Drinking_history *n* (%)					
Drink	38	46.9%	35	62.5%	0.073
Never drinked	43	53.1%	21	37.5%	
Anti-PD-1 treatment line *n* (%)					
First line	42	51.9%	24	42.9%	0.421
Second line	34	42.0%	30	53.6%	
Third line	5	6.2%	2	3.6%	
Treatment type *n* (%)					
Anti-PD-1 monotherapy	8	9.9%	7	12.5%	0.741
Combination therapy					
Anti-PD-1 plus chemotherapy	55	67.9%	37	66.1%	
Anti-PD-1 plus antiangiogenic therapy	18	22.2%	12	21.4%	

ECOG PS: Eastern Cooperative Oncology Group Performance Status; PD-L1: programmed death ligand-1.

**Table 2:** Treatment response in the NLR < 3.23 group and in the NLR ≥ 3.23 group.

Response	NLR < 3.23		NLR ≥ 3.23		*p* value
	*N*	%	*N*	%	
No. patients	81		56		
Response					
CR	2	2.5%	1	1.8%	
PR	27	33.3%	15	26.8%	
SD	41	50.6%	28	50.0%	
PD	11	13.6%	12	21.4%	
ORR					0.377
CR+PR	29	35.8%	16	28.6%	
SD+PD	52	64.2%	40	71.4%	
DCR					0.229
CR+PR+SD	70	86.4%	44	78.6%	
PD	11	13.6%	12	21.4%	

**Table 3:** Univariate analysis and multivariate analysis of progression-free time.

Variable category	Category	Univariate analysis		Multivariate analysis	
		HR (95% CI)	*p* value	HR (95% CI)	*p* value
Gender	Female versus male	0.97 (0.64-1.47)	0.894	0.92 (0.556-1.533)	0.757
Age	⩾59 versus <59	1.29 (0.88-1.88)	0.182	1.33 (0.83-2.11)	0.236
ECOG	⩾2 versus 0-1	1.96 (1.14-3.37)	0.012	2.74 (1.52-4.96)	0.001
Tumor_location	Cardia versus body/fundus versus pylorus	0.89 (0.70-1.10)	0.114	1.07 (0.81-1.40)	0.636
Histological_differentiation	Poorly versus moderately and well	1.44 (0.99-2.08)	0.051	1.54 (1.00-2.37)	0.049
No. of metastasis organs	≥2 versus <2	1.40 (0.91-2.17)	0.119	1.24 (0.78-1.97)	0.370
Liver metastasis	Yes versus no	0.99 (0.68-1.45)	0.966	0.96 (0.61-1.49)	0.847
Smoking_history	Smoke versus never smoked	1.39 (0.97-2.02)	0.072	1.37 (0.83-2.27)	0.223
Drinking_history	Drink versus never drinked	0.84 (0.58-1.22)	0.361	0.95 (0.57-1.58)	0.830
Anti-PD-1 therapy line	First line versus second line and third line	0.61 (0.43-0.89)	0.008	0.65 (0.41-1.04)	0.074
Treatment type	Anti-PD-1 plus chemotherapy versus anti-PD-1 monotherapy or antiangiogenesis	0.67 (0.45-0.94)	0.033	1.12 (0.68-1.87)	0.647
Baseline NLR	<3.23 versus ≥3.23	0.42 (0.29-0.62)	<0.001	0.43 (0.29-0.64)	<0.001

CI: confidence interval; ECOG PS: Eastern Cooperative Oncology Group Performance Status; NLR: neutrophil-to-lymphocyte ratio; HR: hazard ratio.

**Table 4:** Univariate analysis and multivariate analysis of overall survival time.

Variable category	Category	HR (95% CI)	*p* value	HR (95% CI)	*p* value
Gender	Female versus male	0.99 (0.64-1.52)	0.955	0.82 (0.48-1.39)	0.468
Age	⩾59 versus <59	1.29 (0.87-1.89)	0.199	1.34 (0.83-2.18)	0.230
ECOG	⩾2 versus 0-1	1.18 (0.67-2.08)	0.557	1.58 (0.85-2.93)	0.146
Tumor_location	Cardia versus body/fundus versus pylorus	0.89 (0.70-1.12)	0.103	0.99 (0.74-1.33)	0.968
Histological_differentiation	Poorly versus moderately and well	1.92 (1.29-2.86)	0.001	1.89 (1.21-2.94)	0.005
No. of metastasis organs	≥2 versus <2	1.39 (0.87-2.20)	0.156	1.15 (0.71-1.87)	0.564
Liver metastasis	Yes versus no	0.84 (0.57-1.25)	0.396	0.88 (0.54-1.41)	0.584
Smoking_history	Smoke versus never smoked	1.40 (0.96-2.06)	0.077	1.38 (0.78-2.43)	0.271
Drinking_history	Drink versus never drinked	0.73 (0.49-1.07)	0.110	0.73 (0.42-1.26)	0.259
Anti-PD-1 therapy line	First line versus second line and third line	0.551 (0.38-0.80)	0.002	0.64 (0.39-1.06)	0.086
Treatment type	Anti-PD-1 plus chemotherapy versus anti-PD-1 monotherapy or antiangiogenesis	0.49 (0.33-0.73)	<0.001	0.73 (0.43-1.24)	0.249
Baseline NLR	<3.23 versus ≥3.23	0.38 (0.26-0.57)	<0.001	0.34 (0.22-0.52)	<0.001

CI: confidence interval; ECOG PS: Eastern Cooperative Oncology Group Performance Status; NLR: neutrophil-to-lymphocyte ratio; HR: hazard ratio.

## Data Availability

Data are provided in the Supplementary Information files that were submitted alongside the manuscript.

## Supplementary Materials

Supplementary Materials The supplementary data are the complete clinical profiles of the patients involved in the study.
