# Supplementary material for: Neutrophil-to-Lymphocyte Ratio (NLR) Predicts PD-1 Inhibitor Survival in Patients with Metastatic Gastric Cancer
Source: J Immunol Res. 2021 Dec 28;2021:2549295. doi: 10.1155/2021/2549295 (PMC8727102; doi:10.1155/2021/2549295)
Supplement: Supplementary Materials — The supplementary data are the complete clinical profiles of the patients involved in the study. [file 2549295.f1.pdf]

ID号	No	gender	age_	PD-L1	ECOGPS	tumorlocation	differentiation	surgery
Y4153712	1	1	1	1	1	1	2	0
Y4435846	2	1	2	1	0	2	2	1
Y4481284	3	2	1	1	0	2	2	0
Y4559338	4	1	2	1	1	1	2	0
Y4576508	5	2	2	1	0	2	1	1
Y4820332	6	1	2	2	1	2	2	0
C505264	7	2	1	0	0	2	1	0
Y3282094	8	1	1	0	0	2	2	0
Y3302155	9	2	2	0	1	1	1	1
Y3471088	10	1	2	0	0	2	2	0
Y4154876	11	1	1	1	0	1	1	1
Y3231147	12	2	2	0	2	2	1	0
Y4377401	13	1	2	0	0	2	3	0
Y4392907	14	1	1	0	0	1	2	0
Y4350595	15	1	2	0	1	1	2	0
Y4568691	16	1	2	0	0	2	1	0
Y4629919	17	1	1	0	0	3	1	1
Y4670963	18	1	1	0	0	2	1	0
Y3633294	19	1	1	2	0	2	1	0
D667609	20	2	2	2	0	1	1	0
Y4906726	21	1	2	1	1	2	2	0
Y5071634	22	2	2	0	1	2	1	0
Y3247995	23	1	1	2	0	2	1	1
Y1292640	24	1	2	0	0	2	1	1
C505264	25	2	1	0	1	3	1	0
K0128391	26	1	2	0	1	1	2	0
Y0988778	27	1	2	0	1	2	1	0
Y1558948	28	1	1	0	2	2	1	1
Y1728276	29	1	2	0	1	1	2	1
Y2438545	30	1	1	0	1	2	2	1
Y3164696	31	1	2	0	1	1	1	0
Y3420112	32	1	1	0	1	1	2	0
Y3645123	33	1	2	0	1	2	2	0
Y3789342	34	1	1	1	0	2	1	1
Y4442529	35	2	2	2	1	2	1	0
Y5137137	36	1	1	0	0	2	2	1
Y5080793	37	1	2	0	0	1	1	0
Y5167189	38	2	2	2	1	2	1	0
Y5089548	39	1	2	0	1	2	2	0
Y5055713	40	1	1	2	1	2	1	1
Y5287197	41	1	2	1	1	1	3	1
Y2980577	42	2	1	0	0	2	2	1
Y4488463	43	2	2	1	1	2	2	0
Y2261019	44	1	1	0	1	2	1	0
Y5025214	45	1	2	0	1	1	1	0
Y5191789	46	2	1	0	1	2	1	1
Y2481785	47	1	2	0	1	2	2	0
Y5007203	48	1	1	1	1	3	2	1
Y4809653	49	2	1	2	1	2	1	1
Y4645808	50	1	2	0	1	1	2	1
C505264	51	2	1	0	1	2	1	0
Y1327321	52	1	1	0	1	2	2	1

D667609	53	2	2	2	0	1	1	0
C092864	54	2	2	2	2	1	2	1
C505264	55	2	1	0	1	3	1	0
Y0988778	56	1	2	0	1	2	1	0
Y3645123	57	1	2	1	2	2	2	0
Y2481785	58	1	2	0	1	2	3	1
C505264	59	2	1	0	1	2	1	0
Y1327321	60	1	1	0	1	2	2	1
D667609	61	2	2	2	0	1	1	0
C092864	62	2	2	2	2	1	2	1
C505264	63	2	1	0	1	3	1	0
Y0988778	64	1	2	0	1	2	1	0
Y3645123	65	1	2	1	2	2	2	0
Y2481785	66	1	2	0	1	2	2	1
Y1578750	67	1	2	0	2	2	2	0
A634551	68	2	2	0	1	2	1	1
K0185545	69	2	1	0	1	2	1	0
Y3196519	70	2	1	1	1	2	1	0
G165207	71	1	2	2	1	2	1	0
Y1963392	72	1	2	0	1	1	2	1
G158017	73	1	2	2	1	1	1	0
Y3708514	74	1	2	0	2	1	2	0
Y3772555	75	1	2	2	1	2	2	1
Y2077294	76	1	2	2	1	2	3	0
A239481	77	1	1	0	1	2	2	0
A286541	78	1	1	1	1	2	2	1
Y3908399	79	1	2	1	0	1	1	0
F994337	80	2	1	2	0	2	1	1
A831257	81	1	1	0	1	2	2	1
826185	82	2	1	0	1	2	1	1
G183433	83	1	2	1	0	3	2	1
Y4339684	84	1	1	0	1	2	1	1
K0469715	85	1	2	0	1	2	3	0
Y4484338	86	1	1	0	1	3	2	1
G158867	87	1	2	0	1	2	1	1
Y4358337	88	1	1	0	1	2	2	0
Y4903087	89	2	2	2	0	2	1	0
Y4597829	90	2	1	0	1	2	1	1
368492	91	1	2	2	2	2	1	1
C132938	92	1	2	0	1	1	2	1
D111198	93	1	1	2	1	2	1	1
F918783	94	2	1	1	1	2	1	0
F994337	95	1	1	2	2	2	1	0
G108758	96	1	1	0	1	1	1	0
G131031	97	1	1	0	1	2	1	1
K0119532	98	1	2	2	1	2	2	1
K0168554	99	1	2	0	1	2	2	1
Y1045594	100	1	1	2	1	2	2	0
Y1053455	101	1	2	0	1	2	2	1
Y1236445	102	1	2	1	1	2	1	1
Y1309204	103	1	1	0	1	1	1	1
Y1327321	104	1	1	0	1	3	2	1
Y1737537	105	2	1	2	1	2	1	1
Y1963392	106	1	2	0	0	1	2	1

Y2033553	107	2	2	0	0	2	2	1
Y2090079	108	2	2	0	1	1	1	0
Y2305132	109	1	1	0	1	3	1	1
Y2414804	110	1	2	0	1	2	2	1
Y3196519	111	2	1	1	2	2	1	0
Y3654953	112	1	1	0	1	2	2	0
Y3708514	113	1	2	0	2	1	3	0
Y3924085	114	1	2	0	0	3	2	0
Y3981457	115	1	1	0	1	2	1	0
Y4792631	116	1	1	1	1	2	2	0
Y4143354	117	2	2	0	1	1	1	0
Y3259172	118	2	2	1	1	2	2	1
Y5051317	119	1	2	1	0	1	2	0
Y1631605	120	1	2	0	1	2	2	1
Y4272931	121	1	1	0	1	1	1	1
K0144083	122	1	2	0	2	2	2	1
Y0634724	123	1	2	0	0	2	1	1
Y4576508	124	2	2	1	0	2	1	1
Y4629919	125	1	1	0	0	3	1	1
D667609	126	2	2	2	0	1	1	0
K0025599	127	1	2	0	1	1	1	0
Y3424448	128	1	2	2	1	2	1	0
C132938	129	1	2	0	0	1	2	1
Y1963392	130	1	2	0	1	1	2	1
K0025599	131	1	2	0	1	1	1	0
Y3424448	132	1	2	2	1	2	1	0
C132938	133	1	2	0	0	1	2	1
Y3478094	134	1	2	1	1	2	1	1
F586365	135	1	2	2	2	2	2	1
F994337	136	2	1	2	2	2	1	1
Y1963392	137	1	2	0	1	1	2	1

numberofM2	livermeta	smokinghistory	drinkinghistory	Line	eatment_t	wbc	N
2	0	1	1	1	1	4.25	0.738
2	0	1	1	1	2	6.25	0.76
2	1	0	0	1	1	2.12	0.65
2	1	1	1	1	1	8.65	0.676
2	0	0	0	1	1	9.84	0.792
2	1	0	1	1	1	5.22	0.607
2	0	0	0	1	1	4.04	0.665
2	1	1	1	1	1	5.29	0.511
2	0	1	0	1	1	5.44	0.672
2	1	0	0	1	1	5.84	0.677
1	0	1	1	1	1	5.1	0.624
2	0	0	0	1	1	2.17	0.457
2	1	1	1	1	1	5.6	0.592
2	1	0	0	1	1	10.17	0.688
2	1	0	1	1	1	5.35	0.64
2	1	1	1	1	1	5.33	0.692
2	0	0	1	1	1	5.26	0.629
2	1	0	0	1	1	10.17	0.796
2	0	0	0	1	1	6.41	0.713
2	1	0	0	1	1	6.87	0.734
2	1	1	1	1	1	5.39	0.623
2	0	0	0	1	2	5.15	0.747
1	0	1	1	1	1	4.16	0.688
2	0	0	0	1	1	4.69	0.419
2	0	0	0	1	1	5.91	0.665
2	1	1	1	1	2	7.04	0.688
2	0	1	1	1	1	5.95	0.716
2	1	1	1	1	1	4.15	0.643
2	0	1	0	1	2	3.45	0.77
1	0	1	1	1	1	4.15	0.619
2	1	1	1	1	1	4.56	0.648
2	0	1	1	1	1	3.39	0.643
2	1	1	1	1	1	6.71	0.851
2	1	1	1	1	1	5.02	0.391
2	0	0	0	1	1	4.44	0.623
1	1	1	1	1	1	5.3	0.448
1	1	1	1	1	1	7.16	0.589
1	1	0	0	1	1	8.14	0.7088
2	1	0	0	1	1	6.65	0.732
1	1	0	0	1	1	7.73	0.694
1	1	0	0	1	1	3.93	0.456
2	0	0	0	1	1	9.45	0.668
1	1	0	0	1	1	4.38	0.736
2	0	0	1	1	1	4.23	0.645
1	0	0	0	1	1	7.1	0.673
1	0	0	0	1	1	4.5	0.83
1	0	0	0	1	1	6.43	0.592
1	0	1	1	1	1	5.2	0.703
2	0	0	0	1	1	3.02	0.464
1	0	0	1	1	1	10.53	0.603
2	0	0	0	1	1	5.91	0.665
1	1	0	1	1	1	4.52	0.639

2	1	0	0	1	1	6.87	0.805
1	0	0	0	1	1	3.83	0.509
2	0	0	0	1	1	5.91	0.9
2	0	1	0	1	1	5.95	0.717
2	1	1	1	1	1	6.71	0.571
2	0	0	0	1	1	6.43	0.592
2	0	0	0	1	1	5.91	0.665
1	1	0	1	1	1	4.52	0.639
2	1	0	0	1	1	6.87	0.805
1	0	0	0	1	1	3.83	0.509
2	0	0	0	1	1	5.91	0.9
2	0	1	0	1	1	5.95	0.717
2	1	1	1	1	1	6.71	0.571
2	0	0	0	1	1	6.43	0.592
1	1	1	0	2	0	4.27	0.627
2	0	0	0	2	0	14.04	0.806
1	0	0	0	2	0	4.57	0.767
2	0	0	0	2	1	5.62	0.819
1	1	1	0	2	1	4.14	0.721
2	0	0	0	2	1	7.98	0.655
2	1	0	0	2	2	3.03	0.719
2	1	0	0	2	2	4.03	0.948
2	1	1	1	2	2	3.79	0.637
2	0	0	0	2	1	4.97	0.707
1	1	1	1	2	1	5.76	0.6
2	1	0	0	2	1	5.1	0.793
1	1	1	1	2	2	6.34	0.574
2	0	1	1	2	2	2.76	0.488
2	0	0	0	2	1	4.72	0.684
2	0	0	0	2	1	5.3	0.558
2	0	0	1	2	2	3.25	0.616
1	1	0	1	2	2	3.12	0.376
2	1	1	0	2	2	5.28	0.704
2	0	0	1	2	1	4.31	0.708
2	0	0	1	2	1	6.01	0.67
2	0	0	1	2	2	5.4	0.66
1	0	0	0	2	0	3.98	0.452
2	0	0	1	2	2	4.66	0.9
2	0	1	0	2	0	3.48	0.496
2	1	1	1	2	1	6.37	0.731
1	0	1	1	2	0	10.67	0.72
2	0	0	0	2	0	6.32	0.884
2	0	0	0	2	2	2.76	0.488
2	0	1	1	2	2	5.04	0.736
2	0	1	1	2	1	5.55	0.721
2	0	0	0	2	0	5.34	0.71
2	1	0	1	2	1	4.55	0.74
2	0	1	1	2	2	6.58	0.737
2	1	1	1	2	0	4.99	0.823
1	0	1	1	2	2	3.44	0.68
2	0	1	1	2	0	5.16	0.731
2	1	0	1	2	1	4.52	0.642
2	0	0	0	2	0	3.85	0.665
2	1	1	1	2	1	7.98	0.655

1	0	0	0	2	0	2.98	0.616
1	0	0	0	2	1	4.29	0.78
2	0	0	0	2	0	6.13	0.748
2	1	1	0	2	2	14.88	0.955
2	0	0	0	2	1	5.62	0.819
1	0	1	1	2	1	4.09	0.757
2	1	0	0	2	0	4.03	0.711
2	1	0	0	2	2	12.11	0.63
2	0	1	0	2	1	5.79	0.717
1	0	0	0	2	1	6.28	0.777
2	0	1	1	2	2	5.89	0.637
1	0	0	0	2	1	4.58	0.691
2	0	1	1	2	1	9.99	0.63
1	0	0	0	2	1	3.91	0.657
2	0	0	0	2	2	5.94	0.64
1	0	1	1	2	2	5.07	0.587
2	0	0	0	2	2	4.26	0.52
2	0	0	0	2	2	2.77	0.417
2	0	0	1	2	2	3.36	0.565
2	1	0	0	2	1	10.39	0.955
2	0	1	0	2	0	9.96	0.597
2	1	1	0	2	1	4.95	0.721
2	1	0	0	2	1	4.45	0.586
2	1	1	1	2	1	7.98	0.655
2	0	1	0	3	1	10.52	0.597
2	1	1	0	3	2	9.17	0.721
2	1	0	0	3	2	4.34	0.586
1	1	1	1	3	1	4.44	0.657
2	1	1	1	3	1	5.98	0.693
2	0	0	0	3	2	3.05	0.648
2	1	1	1	3	2	6.40983	0.586

L	absoulte N	NLR	NLR_G	response	start_time	end_time	PFS_status
0.188	3.1365	3.925531915	2	3	19-Jan-20	13-Oct-20	1
0.139	4.75	5.467625899	2	2	26-Jul-19	1-Mar-20	1
0.244	1.378	2.663934426	1	2	31-Mar-20	31-Dec-20	0
0.169	5.8474	4	2	4	5-Sep-19	22-Nov-19	1
0.106	7.79328	7.471698113	2	2	14-Sep-19	26-Dec-19	1
0.301	3.16854	2.016611296	1	2	19-Dec-19	6-Jul-20	1
0.232	2.6866	2.86637931	1	2	14-Jan-18	30-Oct-18	1
0.395	2.70319	1.293670886	1	2	23-Mar-18	10-Mar-20	1
0.208	3.65568	3.230769231	2	3	17-Mar-18	1-Sep-18	1
0.197	3.95368	3.436548223	2	2	5-Jun-18	24-Aug-18	1
0.28	3.1824	2.228571429	1	3	15-May-19	23-Apr-20	1
0.417	0.99169	1.095923261	1	4	4-Jun-19	16-Jul-19	1
0.309	3.3152	1.915857605	1	2	25-Jun-19	10-Mar-20	1
0.212	6.99696	3.245283019	2	2	12-Jul-19	15-Mar-20	1
0.264	3.424	2.424242424	1	3	3-Jul-19	1-Feb-20	1
0.186	3.68836	3.720430108	2	2	23-Sep-19	1-Apr-20	1
0.254	3.30854	2.476377953	1	3	25-Oct-19	6-Mar-20	1
0.118	8.09532	6.745762712	2	4	24-Oct-19	10-Dec-19	1
0.222	4.57033	3.211711712	2	2	16-May-19	13-Dec-20	0
0.177	5.04258	4.146892655	2	2	18-Dec-19	22-Apr-20	1
0.282	3.35797	2.209219858	1	2	18-Mar-20	31-Dec-20	0
0.171	3.84705	4.368421053	2	4	23-Jul-20	18-Sep-20	1
0.2	2.86208	3.44	2	3	9-Mar-19	1-Jun-19	0
0.465	1.96511	0.901075269	1	3	22-Nov-18	2-Jul-19	1
0.232	3.93015	2.86637931	1	2	24-Dec-17	11-Oct-18	1
0.207	4.84352	3.323671498	2	4	7-Sep-17	23-Nov-17	1
0.205	4.2602	3.492682927	2	2	14-Dec-17	2-Apr-18	1
0.254	2.66845	2.531496063	1	4	29-Jun-16	15-Jul-16	1
0.131	2.6565	5.877862595	2	3	31-Dec-16	5-Jun-17	1
0.259	2.56885	2.38996139	1	3	8-Nov-18	24-Oct-19	1
0.251	2.95488	2.581673307	1	3	13-Jan-18	28-Jul-18	1
0.265	2.17977	2.426415094	1	2	4-Jun-18	9-May-19	1
0.094	5.71021	9.053191489	2	3	17-Sep-18	18-Jan-19	1
0.525	1.96282	0.744761905	1	2	9-Nov-18	1-Apr-19	1
0.239	2.76612	2.606694561	1	2	16-Jul-19	1-Mar-20	1
0.347	2.3744	1.291066282	1	3	12-Aug-20	9-Dec-20	1
0.233	4.21724	2.527896996	1	2	18-Jul-20	31-Dec-20	0
0.189	5.769632	3.75026455	2	3	25-Aug-20	31-Dec-20	0
0.164	4.8678	4.463414634	2	3	24-Jul-20	5-Nov-20	1
0.226	5.36462	3.07079646	2	4	7-Aug-20	20-Sep-20	1
0.374	1.79208	1.219251337	1	3	16-Oct-20	31-Dec-20	0
0.232	6.3126	2.879310345	1	2	28-May-20	31-Dec-20	0
0.202	3.22368	3.643564356	2	3	23-Apr-20	31-Dec-20	0
0.244	2.72835	2.643442623	1	3	15-Sep-20	31-Dec-20	0
0.211	4.7783	3.18957346	2	2	11-Jun-20	3-Dec-20	0
0.356	3.735	2.331460674	1	3	31-Aug-20	30-Nov-20	1
0.319	3.80656	1.855799373	1	3	10-Aug-19	17-Nov-20	1
0.156	3.6556	4.506410256	2	3	24-Sep-20	31-Dec-20	1
0.46	1.40128	1.008695652	1	3	22-Jan-20	31-Dec-20	0
0.247	6.34959	2.441295547	1	2	29-Dec-19	31-Dec-20	0
0.232	3.93015	2.86637931	1	2	24-Dec-17	25-Oct-18	1
0.243	2.88828	2.62962963	1	3	23-Sep-15	12-Dec-20	1

0.098	5.53035	8.214285714	2	2	18-Dec-19	7-Mar-20	1
0.405	1.94947	1.256790123	1	2	4-Dec-18	3-Sep-19	1
0.039	5.319	23.07692308	2	2	24-Dec-17	22-Nov-18	1
0.205	4.26615	3.497560976	2	2	14-Dec-17	2-Apr-18	1
0.298	3.83141	1.916107383	1	3	17-Sep-18	29-Mar-19	1
0.319	3.80656	1.855799373	1	3	10-Aug-19	20-Oct-20	1
0.232	3.93015	2.86637931	1	2	24-Dec-17	25-Oct-18	1
0.243	2.88828	2.62962963	1	3	23-Sep-15	12-Dec-20	1
0.098	5.53035	8.214285714	2	2	18-Dec-19	7-Mar-20	1
0.405	1.94947	1.256790123	1	2	4-Dec-18	3-Sep-19	1
0.039	5.319	23.07692308	2	2	24-Dec-17	22-Nov-18	1
0.205	4.26615	3.497560976	2	2	14-Dec-17	2-Apr-18	1
0.298	3.83141	1.916107383	1	3	17-Sep-18	29-Mar-19	1
0.319	3.80656	1.855799373	1	3	10-Aug-19	20-Oct-20	1
0.276	2.67729	2.27173913	1	3	28-Feb-18	6-Sep-18	1
0.091	11.31624	8.857142857	2	3	14-Jul-17	7-Sep-17	1
0.183	3.50519	4.191256831	2	4	29-Jul-18	26-Oct-18	1
0.137	4.60278	5.97810219	2	3	23-Aug-18	11-Dec-18	1
0.164	2.98494	4.396341463	2	4	25-Jul-18	12-Sep-18	1
0.227	5.2269	2.885462555	1	2	19-Oct-18	24-Apr-19	1
0.188	2.17857	3.824468085	2	3	11-Apr-19	5-Sep-20	1
0.032	3.82044	29.625	2	4	15-Nov-18	15-Jan-19	1
0.253	2.41423	2.517786561	1	3	6-Feb-20	4-Dec-20	1
0.209	3.51379	3.38277512	2	3	31-Jan-19	2-Apr-19	1
0.3	3.456	2	1	3	17-Jul-19	11-Nov-19	1
0.111	4.0443	7.144144144	2	2	22-Dec-18	25-Apr-19	1
0.245	3.63916	2.342857143	1	3	26-Oct-19	1-Feb-20	1
0.38	1.34688	1.284210526	1	3	30-Dec-18	21-Aug-19	1
0.212	3.22848	3.226415094	2	4	21-Jul-19	19-Sep-19	1
0.347	2.9574	1.608069164	1	2	17-Jun-20	31-Dec-20	0
0.292	2.002	2.109589041	1	3	29-May-20	7-Dec-20	0
0.519	1.17312	0.724470135	1	3	9-Nov-19	14-Feb-20	1
0.181	3.71712	3.889502762	2	3	8-Nov-19	1-Apr-20	1
0.157	3.05148	4.50955414	2	2	16-Aug-19	**12-Feb-20**	**1**
0.21	4.0267	3.19047619	2	3	13-Mar-19	3-Oct-19	1
0.215	3.564	3.069767442	2	3	28-Dec-19	1-Mar-20	1
0.427	1.79896	1.058548009	1	3	26-Sep-19	13-Jan-20	1
0.071	4.194	12.67605634	2	3	24-Apr-20	15-Sep-20	1
0.416	1.72608	1.192307692	1	4	7-Dec-18	18-Jan-19	1
0.181	4.65647	4.038674033	2	4	27-May-16	25-Jun-16	1
0.17	7.6824	4.235294118	2	4	6-Nov-18	7-Dec-18	1
0.244	5.58688	3.62295082	1	4	28-Apr-16	5-May-16	1
0.388	1.34688	1.257731959	1	3	30-Dec-18	4-Jul-19	1
0.113	3.70944	6.513274336	2	3	23-Oct-17	12-Dec-17	1
0.15	4.00155	4.806666667	2	4	4-Jan-19	1-Mar-19	1
0.137	3.7914	5.182481752	2	4	1-Jul-17	19-Jul-17	1
0.198	3.367	3.737373737	2	3	6-Dec-17	26-Jan-18	1
0.169	4.84946	4.360946746	2	3	20-Nov-15	18-Aug-16	1
0.058	4.10677	14.18965517	2	3	2-Dec-15	1-Mar-16	1
0.218	2.3392	3.119266055	2	3	23-Jul-18	1-Dec-18	1
0.133	3.77196	5.496240602	2	3	9-Jan-18	9-May-18	1
0.232	2.90184	2.767241379	1	3	23-Sep-15	31-Dec-20	0
0.262	2.56025	2.538167939	1	4	28-Aug-16	21-Oct-16	1
0.227	5.2269	2.885462555	1	2	19-Oct-18	26-Aug-19	1

0.273	1.83568	2.256410256	1	3	30-Sep-18	5-Aug-19	1
0.114	3.3462	6.842105263	2	3	20-Jun-17	18-Oct-17	1
0.159	4.58524	4.704402516	2	3	20-Jul-17	27-Feb-18	1
0.012	14.2104	79.58333333	2	4	4-Jul-17	4-Nov-17	1
0.137	4.60278	5.97810219	2	3	23-Aug-18	1-Nov-18	1
0.057	3.09613	13.28070175	2	3	4-Nov-18	1-Mar-19	1
0.223	2.86533	3.188340807	2	3	27-Oct-18	15-Jan-19	1
0.251	7.6293	2.509960159	1	3	15-Mar-19	15-Jul-19	1
0.193	4.15143	3.715025907	2	3	11-Jan-19	18-Apr-19	1
0.153	4.87956	5.078431373	2	2	13-Feb-20	23-Dec-20	0
0.202	3.75193	3.153465347	2	3	6-Jan-20	31-Dec-20	0
0.175	3.16478	3.948571429	2	1	15-Nov-19	11-Nov-20	1
0.258	6.2937	2.441860465	1	3	15-Aug-20	2-Nov-20	1
0.225	2.56887	2.92	1	4	14-Aug-19	12-Oct-19	1
0.224	3.8016	2.857142857	1	3	8-Feb-20	6-Apr-20	1
0.302	2.97609	1.943708609	1	3	1-Jun-20	11-Sep-20	1
0.25	2.2152	2.08	1	2	29-Jun-20	31-Dec-20	0
0.371	1.15509	1.123989218	1	2	28-Dec-19	28-Jul-20	1
0.277	1.8984	2.039711191	1	3	6-Mar-20	14-Oct-20	1
0.039	9.92245	24.48717949	2	3	22-Apr-20	4-Jul-20	1
0.289	5.94612	2.065743945	1	1	14-Jul-17	29-Jan-19	1
0.164	3.56895	4.396341463	2	3	25-Jul-18	12-Nov-18	1
0.337	2.6077	1.738872404	1	3	8-Mar-16	26-May-16	1
0.227	5.2269	2.885462555	1	2	19-Oct-18	26-Aug-19	1
0.289	6.28044	2.065743945	1	4	20-Feb-19	1-Apr-19	1
0.164	6.61157	4.396341463	2	3	14-Sep-18	1-Jan-19	1
0.337	2.54324	1.738872404	1	4	7-Jun-16	25-Jun-20	1
0.214	2.91708	3.070093458	2	1	21-Nov-18	30-Mar-20	1
0.191	4.14414	3.628272251	2	3	8-Apr-16	8-Jun-16	1
0.233	1.9764	2.78111588	1	4	21-Aug-19	7-Jan-20	1
0.31	3.7561602	1.890322581	1	2	24-Apr-19	26-Aug-19	1

PFS	PFS_duratior	death_time	OS_STATUS	OS	S_duration
268	9.6	30-Dec-20	1	621	22.2
219	7.8	1-Jun-20	1	311	11.1
275	9.8	31-Dec-20	0	519	18.5
78	2.8	1-Mar-20	1	178	6.4
103	3.7	1-Sep-20	1	353	12.6
200	7.1	31-Dec-20	0	378	13.5
289	10.3	22-Nov-18	1	312	11.1
718	25.6	31-Dec-20	0	1014	36.2
168	6.0	12-Feb-19	1	332	11.9
80	2.9	4-Mar-19	1	272	9.7
344	12.3	31-Dec-19	1	230	8.2
42	1.5	1-Sep-19	1	89	3.2
259	9.3	1-Jun-20	1	342	12.2
247	8.8	1-Oct-20	1	447	16.0
213	7.6	1-May-20	1	303	10.8
191	6.8	31-Dec-20	0	465	16.6
133	4.8	31-Dec-20	0	433	15.5
47	1.7	16-Oct-20	1	358	12.8
577	20.6	31-Dec-20	1	595	21.3
126	4.5	1-Oct-20	1	288	10.3
288	10.3	31-Dec-20	0	288	10.3
57	2.0	18-Sep-20	1	57	2.0
84	3.0	1-Aug-20	1	511	18.3
222	7.9	2-Jul-19	1	222	7.9
291	10.4	1-Feb-19	1	404	14.4
77	2.8	1-Jan-18	1	116	4.1
109	3.9	18-May-18	1	155	5.5
16	0.6	31-Jul-16	1	32	1.1
156	5.6	28-Jul-17	1	209	7.5
350	12.5	31-Dec-20	1	784	28.0
196	7.0	30-Mar-19	1	441	15.8
339	12.1	1-Mar-20	1	636	22.7
123	4.4	1-Jun-20	1	623	22.3
143	5.1	1-Oct-19	1	326	11.6
229	8.2	1-Apr-20	1	260	9.3
119	4.3	31-Dec-20	0	141	5.0
166	5.9	31-Dec-20	1	166	5.9
128	4.6	1-Jan-21	0	129	4.6
104	3.7	31-Dec-20	1	160	5.7
44	1.6	1-Jan-21	1	147	5.3
76	2.7	31-Dec-20	0	76	2.7
217	7.8	1-Jan-21	0	218	7.8
252	9.0	2-Jan-21	0	254	9.1
107	3.8	3-Jan-21	0	110	3.9
175	6.3	31-Dec-20	0	203	7.3
91	3.3	7-Jan-21	1	129	4.6
465	16.6	1-Feb-21	1	541	19.3
98	3.5	1-Feb-21	1	130	4.6
344	12.3	31-Dec-20	0	344	12.3
368	13.1	31-Dec-20	0	368	13.1
305	10.9	1-Feb-19	1	404	14.4
1907	68.1	31-Dec-20	1	1926	68.8

80	2.9	1-Sep-20	1	258	9.2
273	9.8	31-Dec-20	0	758	27.1
333	11.9	23-Dec-18	1	364	13.0
109	3.9	14-May-18	1	151	5.4
193	6.9	1-Oct-19	1	379	13.5
437	15.6	31-Dec-20	0	509	18.2
305	10.9	1-Feb-19	1	404	14.4
1907	68.1	31-Dec-20	1	1926	68.8
80	2.9	1-Sep-20	1	258	9.2
273	9.8	31-Dec-20	0	758	27.1
333	11.9	23-Dec-18	1	364	13.0
109	3.9	14-May-18	1	151	5.4
193	6.9	1-Oct-19	1	379	13.5
437	15.6	31-Dec-20	0	509	18.2
190	6.8	1-Jan-19	1	307	11.0
55	2.0	17-Sep-17	1	65	2.3
89	3.2	1-Jan-19	1	156	5.6
110	3.9	1-Jan-19	1	131	4.7
49	1.8	1-Feb-19	1	191	6.8
187	6.7	1-Mar-20	1	499	17.8
513	18.3	21-Nov-19	1	224	8.0
61	2.2	15-Jan-19	1	61	2.2
302	10.8	31-Dec-20	1	329	11.8
61	2.2	2-May-19	1	91	3.3
117	4.2	20-Jan-20	1	187	6.7
124	4.4	1-Feb-20	1	406	14.5
98	3.5	1-Apr-20	1	158	5.6
234	8.4	15-Jun-20	1	533	19.0
60	2.1	1-Feb-20	1	195	7.0
197	7.0	31-Dec-20	0	197	7.0
192	6.9	31-Dec-20	0	216	7.7
97	3.5	1-May-20	1	174	6.2
145	5.2	1-May-20	1	175	6.3
180	6.4	1-Apr-20	1	229	8.2
204	7.3	1-Apr-20	1	385	13.8
64	2.3	1-Aug-20	1	217	7.8
109	3.9	1-Oct-20	1	371	13.3
144	5.1	1-Oct-20	1	160	5.7
42	1.5	18-Jan-19	1	42	1.5
29	1.0	26-Jun-16	1	30	1.1
31	1.1	31-Dec-18	1	55	2.0
7	0.3	16-Sep-16	1	141	5.0
186	6.6	15-Jun-20	1	533	19.0
50	1.8	1-Mar-18	1	129	4.6
56	2.0	1-May-19	1	117	4.2
18	0.6	1-Oct-17	1	92	3.3
51	1.8	26-Jan-18	1	51	1.8
272	9.7	1-Dec-16	1	377	13.5
90	3.2	11-Nov-16	1	345	12.3
131	4.7	1-Feb-19	1	193	6.9
120	4.3	1-Jul-18	1	173	6.2
1926	68.8	31-Dec-20	0	1926	68.8
54	1.9	21-Oct-17	1	419	15.0
311	11.1	1-Mar-20	1	499	17.8

309	11.0	1-Jul-19	1	274	9.8
120	4.3	1-Jan-18	1	195	7.0
222	7.9	31-Mar-18	1	254	9.1
123	4.4	15-Nov-17	1	134	4.8
70	2.5	1-Jan-19	1	131	4.7
117	4.2	1-May-19	1	178	6.4
80	2.9	15-Jan-19	1	80	2.9
122	4.4	15-Aug-19	1	153	5.5
97	3.5	1-May-19	1	110	3.9
314	11.2	31-Dec-20	0	322	11.5
360	12.9	31-Dec-20	0	360	12.9
362	12.9	31-Dec-20	0	412	14.7
79	2.8	31-Jan-21	1	169	6.0
59	2.1	1-Jan-20	1	140	5.0
58	2.1	1-Jul-20	1	144	5.1
102	3.6	1-Dec-20	1	183	6.5
185	6.6	31-Dec-20	1	185	6.6
213	7.6	1-Sep-20	1	248	8.9
222	7.9	31-Dec-20	0	300	10.7
73	2.6	1-Oct-20	1	162	5.8
564	20.1	1-Apr-19	1	626	22.4
110	3.9	1-Jan-19	1	160	5.7
79	2.8	25-Jun-20	1	1570	56.1
311	11.1	1-Mar-20	1	499	17.8
40	1.4	1-Apr-19	1	40	1.4
109	3.9	1-Jan-19	1	109	3.9
1479	52.8	25-Jun-20	1	1479	52.8
495	17.7	31-Dec-20	0	771	27.5
61	2.2	18-Jul-16	1	101	3.6
139	5.0	16-Jun-20	1	300	10.7
124	4.4	1-Mar-20	1	312	11.1
